# A review of transgenic animal techniques and their applications

**DOI:** 10.1186/s43141-023-00502-z

**Published:** 2023-05-09

**Authors:** W. M. E. Shakweer, A. Y. Krivoruchko, Sh.M. Dessouki, A. A. Khattab

**Affiliations:** 1 Animal Production Department, Agricultural and Biological Research Institute, National Research Centre, 33 El-Buhouth Street, Dokki, Cairo, 12622 Egypt; 2 Genetic and Biotechnology Department, All-Russian Research Institute of Sheep and Goat Breeding, Stavropol, Russia; 3 Department of Animal Production, Faculty of Agriculture, Cairo University, 7 Gamaa Street, Giza, 12613 Egypt; 4 Genetics and Cytology Department, Biotechnology Research Institute, National Research Centre, 33 El-Buhouth Street, Dokki, Cairo, 12622 Egypt

**Keywords:** Transgenic animals, Vectors, DNA microinjection, Sperm-mediated gene transfer, Somatic cell nuclear transfer, Xenotransplantation

## Abstract

Nowadays, breakthroughs in molecular biology are happening at an unprecedented rate. One of them is the ability to engineer transgenic animals. A transgenic animal is one whose genome has been changed to carry genes from another species or to use techniques for animal genome editing for specific traits. Animal features can be changed by purposefully altering the gene (or genes). A mouse was the first successful transgenic animal. Then pigs, sheep, cattle, and rabbits came a few years later. The foreign-interested genes that will be used in animal transgenic techniques are prepared using a variety of methods. The produced gene of interest is placed into a variety of vectors, including yeast artificial chromosomes, bacterial plasmids, and cosmids. Several techniques, including heat shock, electroporation, viruses, the gene gun, microinjection, and liposomes, are used to deliver the created vector, which includes the interesting gene, into the host cell. Transgenesis can be carried out in the gonads, sperm, fertilized eggs, and embryos through DNA microinjection, retroviruses, stem cells, and cloning. The most effective transgenic marker at the moment is fluorescent protein. Although transgenesis raises a number of ethical concerns, this review concentrates on the fundamentals of animal transgenesis and its usage in industry, medicine, and agriculture. Transgenesis success is confirmed by the integration of an antibiotic resistance gene, western and southern blots, PCR, and ELISA. If technology solves social and ethical problems, it will be the most promising in the future.

## Background

The transgenesis technique involves the introduction of foreign DNA sequences into the genome of transfected cells and ensuring that the DNA sequences are integrated and transmitted to the offspring [1]. Greater prolificacy and reproductive performance, improved feed utilization and growth rate, improved carcass composition, improved milk production and/or compositions, and increased disease resistance are some of the practical applications of transgenesis in animal production. Growth hormone is one of the most important candidate genes used to produce transgenic farm animals to increase their growth rate and milk production [2–4]. In germ-line gene transfer, the parents’ egg and sperm cells are altered in order to pass the alterations on to the progeny of the transformed species [5–7]. Nowadays, the gene constructs have now been introduced into the majority of food animals, including cattle, sheep, goats, pigs, rabbits, chickens, and fish [8–11]. The stable insertion of the gene into the germ line has been a great technological achievement in agriculture. Animals with large transgenes are helpful for biotechnology and genetic research, such as the characterization and modulation of large single-gene and polygenic features [3, 4]. As a result, the focus of this review will be on the most important animal transgenesis procedures.

## Approaches to generate transgenic animals

**Fig. 1:**
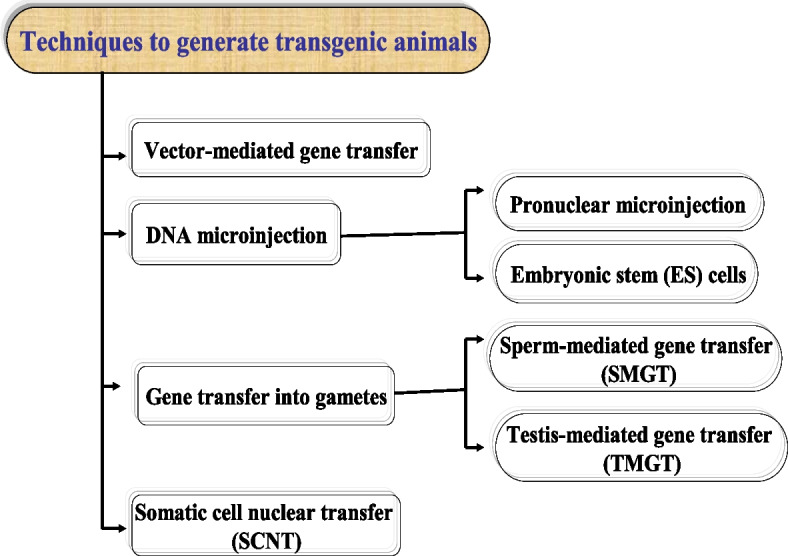
Main techniques used to generate transgenic animals

Various methods for producing transgenic animals have been developed during the last few decades. Many sequences have been determined as a result of gene sequencing, bringing knowledge of promoters and genes of relevance to many species. The advent of genomics, proteomics, and a new generation of reproductive biotechnologies all point to successful transgenic applications in domestic animals. The procedures and methodologies used in the creation of a transgenic animal are determined by the animal’s intended use. Many transgenic animal models have been developed to research gene function, serve as bioreactors, and serve as models for novel animal breeding techniques [1]. The primary approaches utilized to create transgenic animals are shown in Fig. 1. There are three types of foreign DNA transfer techniques: DNA microinjection into pronuclei, mass gene transfer using gametes, and somatic cell nuclear transfer (SCNT).

### Vector-mediated gene transfer

The term “cloning vector” refers to a short amount of DNA with foreign DNA that has the capacity to reproduce itself for use in transferring or propagating in an organism. Vectors increase the probability of gene expression [12]. The various accessible vectors have been developed to hold DNA of various lengths. Plasmids, cosmids, the P1 phage, BACs (bacterial artificial chromosomes), and YACs (yeast artificial chromosomes) may each hold 20 kilobytes (kb), 40 kb, 90 kb, 200 kb, and 1000 kb of DNA. Viruses have the ability to deliver their genome into cells efficiently. Researchers were motivated by this discovery to consider employing viral genomes as foreign DNA vectors [13]. Various forms of viral vectors are now being used or investigated, including the following:

#### Retroviral vectors

They are RNA viruses that can generate DNA from RNA using reverse-transcriptase enzymes. They can copy themselves when a cell divides by integrating into the host DNA [5, 14, 15]. Recently, retroviral vectors were used to allow for the integration of a foreign gene into the host genome. They can carry up to 7 to 8 kb from foreign genes, but at the same time, this may not be enough for long genes or structures that require extensive regulatory sequences for transcription [5, 13].

#### Adeno-associated virus (AAV) vectors

Adeno-associated virus (AAV) was initially detected in human tissues in the mid-1960s from laboratory adenovirus (AdV) preparations [15, 16]. A few research groups set out to grasp basic AAV biology out of pure scientific interest and without recognizing its enormous potential as a human gene therapy platform [16–18]. Several fundamental characteristics of the virus were defined during the first 15–20 years of research, including its genome layout and composition [19], DNA replication and transcription [20], infectious latency [21], and virion assembly [22]. The successful cloning of the wild-type AAV2 sequence into plasmids, which permitted genetic studies [23], and the sequencing of the full AAV2 genome [24], was made possible by these accomplishments. These early studies provided crucial information that led to the development of AAV as a gene delivery vehicle, which could carry about 10 kb of foreign DNA.

#### Adenoviral vectors

Adenoviral vectors are double-stranded DNA vectors that are not enveloped. Adenoviral vectors are extensively utilized as research tools in vitro and in small animal models due to their relatively easy manufacture and high levels of transgene expression [25]. Adenovirus vectors (AdV) are extremely strong gene transfer vehicles, with applications capable of holding up to 10 kb of foreign DNA. The elimination of structural genes such as gag, pol, and env, which aid in the assembly of viral particles by the retrovirus, is a common change in this type of vector [26].

## DNA microinjection technique

### Pronuclear DNA microinjection

**Fig. 2:**
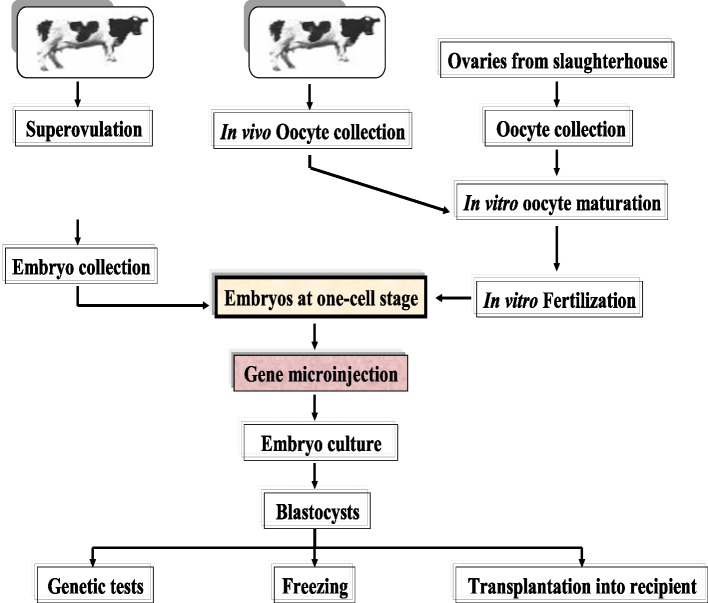
Showing the pronuclear DNA microinjection technique in cow

A variety of approaches can be used to make transgenic animals. The most common method used to date is the microinjection of genes into the pronuclei of zygotes. In the 1980s, this method was first used on rabbits, pigs, and sheep and thereafter on goats and cows. However, the usefulness of this approach for domestic animals, is still limited [27]. The major drawback of this method is that some copies of the foreign gene are randomly integrated into the host genome, causing transgene and host gene expression to be disrupted. The experiment requires a large number of embryos in the pronucleus stage. Thus, the average progeny obtained ranges from 1 to 4% when 500 to 5000 copies of foreign DNA are introduced into the pronuclei. This indicates that from a hundred injected cells, only 1–4 transgenic mice are produced. In cattle, the success rate is the lowest. Because of the low rate of integration of injected DNA into the genome and the restricted embryonic survival, producing transgenic cattle via pronuclear microinjection of DNA into fertilized zygotes is difficult. The pronuclear DNA microinjection technique in cows is shown in Fig. 2. Pronuclear DNA microinjection has long been the most effective method for producing transgenic offspring in pigs; yet, even in this species, the efficacy of transgenic offspring production is limited, with only 1% of DNA-injected embryos resulting in transgenic animals [28]. The success rate of the pronuclear DNA microinjection technique is low and varies between species [29]. The reasons for this divergence are unknown, although they are most likely related to changes in the DNA repair mechanism or the host genome’s intrinsic DNA integration process. Furthermore, low transgenesis efficiency in domestic animals may be attributable to exogenous DNA purity, the method used to create the artificial molecule (promoters and coding sections), and other cellular machinery-related characteristics [29].

### Embryonic stem (ES) cells

**Fig. 3:**
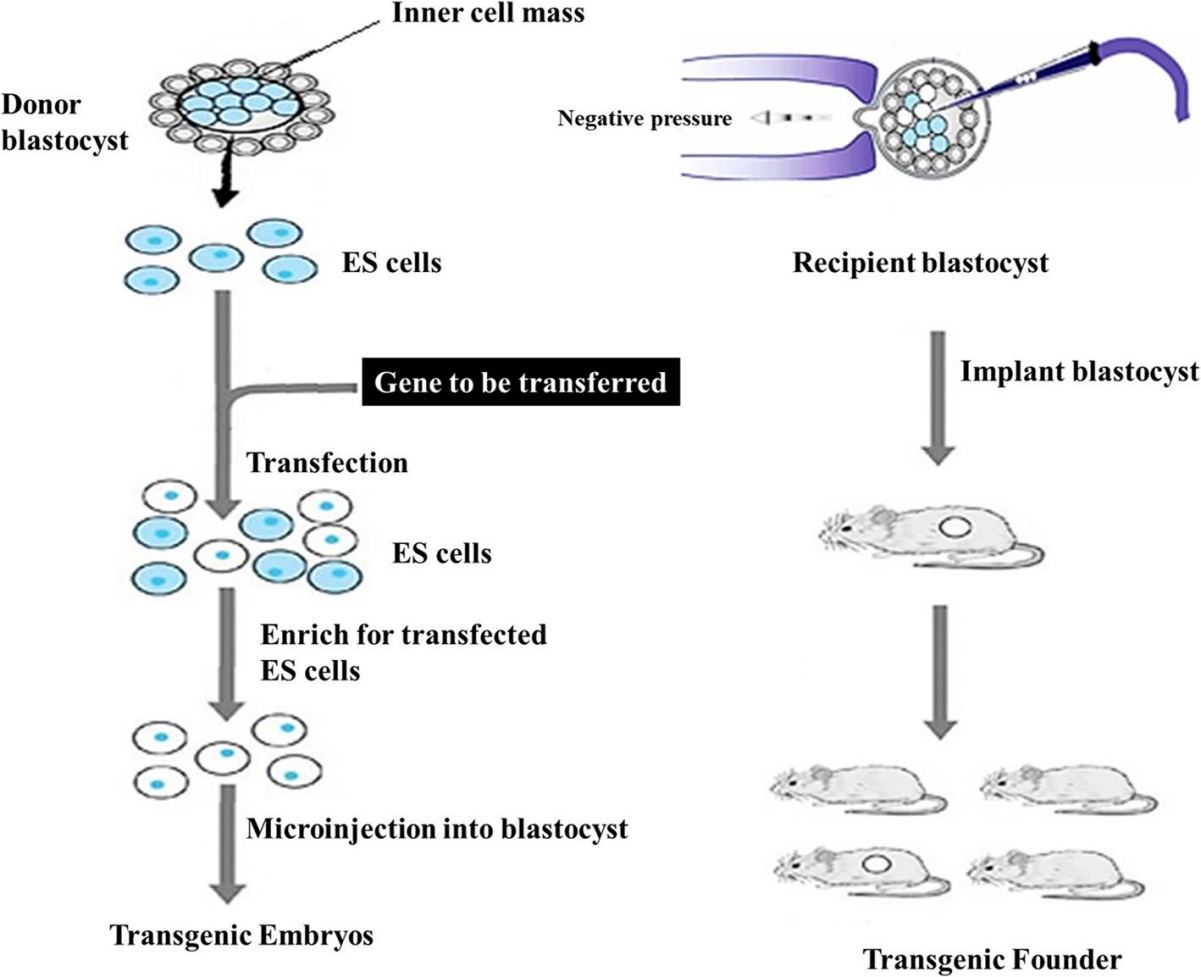
The DNA microinjection technique using ES cells

**Fig. 4:**
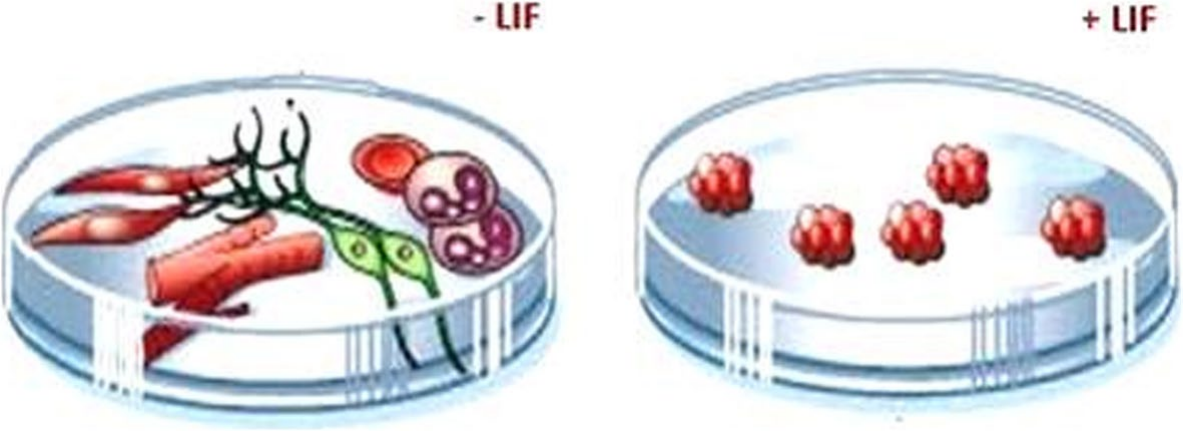
In vitro culture of embryonic stem (ES) cells

The properties of stem cells are undifferentiated cells, undifferentiated cells, and undifferentiated cells. 2. Have the ability to develop into any type of cell (including somatic and germ cells), leading to the production of a full organism. Embryonic stem cells have been developed in vitro for a long period of time [30]. The appropriate DNA sequence is inserted into an in vitro culture of embryonic stem (ES) cells using homologous recombination. Foreign DNA can be introduced into ES cells, and utilizing a selection gene, clones carrying the foreign gene can be generated. These cells can be used to make transgenic chimeric mice (Fig. 3). In these animals, the transgene is mosaic [31]. In the laboratory, when a leukemia inhibitory factor (LIF) is given to the culture, the stem cells stay undifferentiated. Because LIF is absent, ES cells can develop into a variety of tissues on their own (Fig. 4).

## Gene transfer into gametes

### Sperm-mediated gene transfer technique (SMGT)

**Fig. 5:**
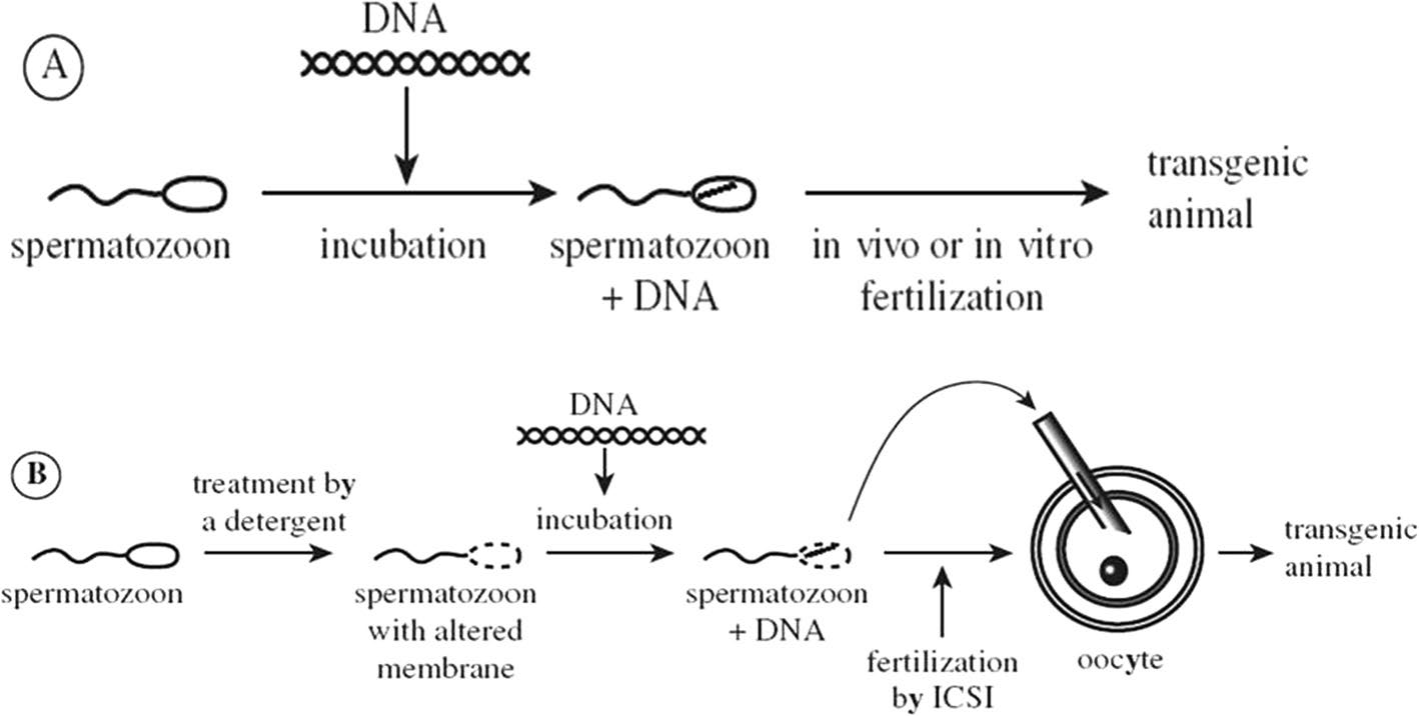
The sperm-mediated gene transfer technique

The first indication that foreign DNA might be integrated into untreated sperm was given by Brackett et al. [32]. Lavitrano et al. [33] demonstrated for the first time that (a) mouse epididymal sperm can spontaneously incorporate plasmid DNA molecules, (b) genetically modified offspring can be generated by in vitro fertilization procedures using plasmid-containing sperm cells, (c) exogenous DNA sequences are expressed in the progenitors, and (d) sperm-carried exogenous DNA is incorporated into the fertilized ovum (Fig. 5).

Transgenic mice, rabbits, pigs, sheep, cows, chickens, and fish have been created by incubating sperm cells with foreign DNA and fertilizing them in vitro or in vivo [9, 10]. Furthermore, this operation does not necessitate any special equipment or skills, and it may be carried out in the field. Another fascinating feature of using sperm as DNA vectors is the concept of mass transgenesis [9, 10]. In subsequent studies, the successful introduction of the exogenous GH expression vector into the sperm head allowed for the production of GH-transgenic sheep characterized by a high growth rate in order to reduce the meat shortage in Egypt [10]. The main binding site of foreign DNA in mouse sperm is mediated by a complex structure of molecules from the class 2 major histocompatibility complex, which is found in the posterior area of the sperm head, according to Wu et al. [34]. In the mouse seminal plasma, researchers discovered two components: a DNase from the seminal vesicle and a variety of foreign DNA-binding proteins from the prostate. Exogenous DNA sequestration has been shown to be inhibited by these components [35, 36].

SMGT is employed in domestic animals such as cattle and pigs by taking advantage of the farmers’ standard artificial insemination (AI) method [37]. Fresh semen is taken from donor animals and cleaned several times before being centrifuged to remove seminal plasma. Animal artificial insemination, incubation of sperm cell suspensions with foreign plasmid DNA (about 1 h at 18), and dilution in suitable extender dimethyl sulfoxide (DMSO) and Triton X-100, a mild polar detergent, were used to improve DNA uptake in sperm [9, 10]. The sperm membrane was destabilized as a result, allowing foreign DNA full access to the sperm. Also, using sperm freezing and thawing, similar findings have been produced [38].

Another fascinating alternative method is intracytoplasmic sperm injection, which involves injecting sperm that has been treated and incubated with foreign DNA directly into the oocyte (ICSI). ICSI has been used to successfully transfer lengthy pieces of DNA in mice, as well as in yeast, bacteria, and other artificial chromosomal constructs (YACs, BACs, and MACs) [39, 40]. Chang et al. [41] describe an intriguing method for producing transgenic animals that involves incubating sperm cells with tagged foreign DNA and monoclonal antibodies (mAb C). mAb C is a simple protein that attaches to DNA via ionic interactions, allowing foreign DNA to be connected to sperm selectively. The surface antigen on the sperm of all studied species, including pig, mouse, chicken, cow, goat, sheep, and human, is reactive to this linker protein. It is worth noting that foreign DNA uptake mediating mechanisms are an important aspect of the biology of sexually reproducing organisms [36].

### In vitro sperm precursors

**Fig. 6:**
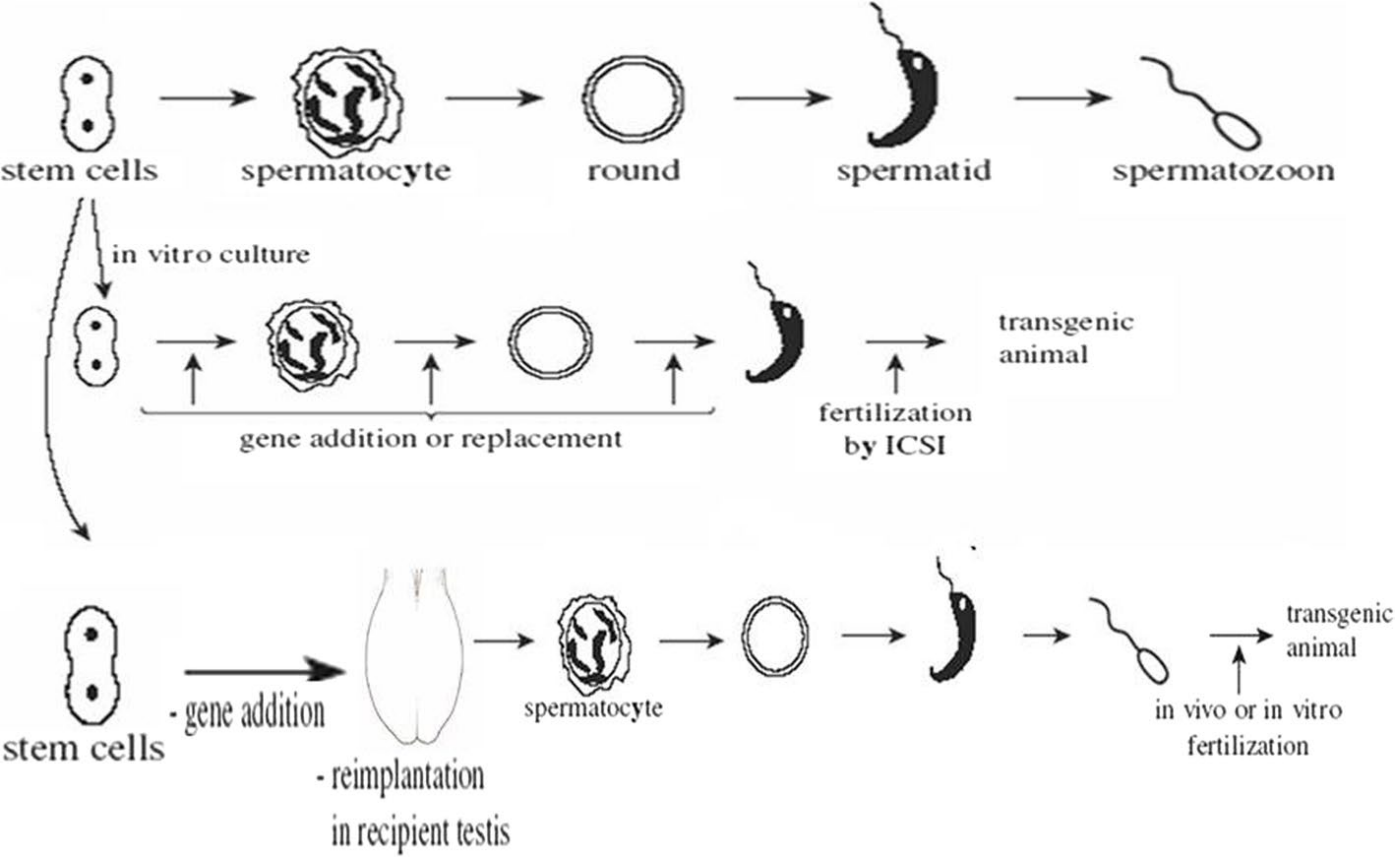
The sperm-mediated gene transfer technique (in vitro and in vivo sperm precursors)

The production of mature sperm from stem cells is known to occur at various stages of differentiation (Fig. 6). Sperm stem cells can be extracted, grown in vitro for a brief time, and then transferred into an adoptive testis. The transplanted cells continue to differentiate, eventually producing functioning sperm. Treatment of recipient males with busulfan, a medication that prevents testis stem cell development, dramatically enhanced the amount of sperm produced by the transplanted stem cells. This approach has been used to successfully transfer genes into stem cells while they are being cultured. This was accomplished with the use of a powerful retroviral vector. Transgenic mice were created at a rate of up to 4% when stem cells were transplanted into busulfan-treated recipient males. This technology could be used to investigate the biological effects of genes during the maturation of sperm stem cells and to create transgenic animals. Extrapolation to species larger than mice is unlikely to be successful. Indeed, more highly altered cells appear to be required to raise the chances of colonizing testis at a significant pace.

### Testis-mediated gene transfer technique (TMGT)

**Fig. 7:**
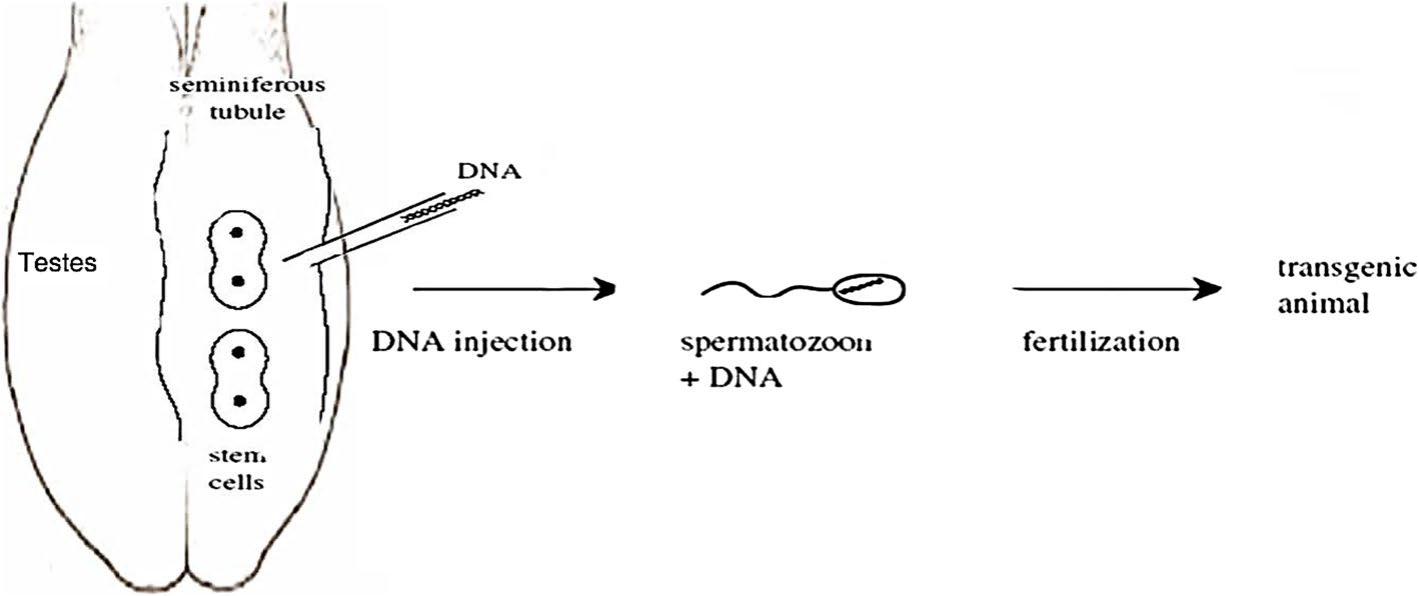
The sperm-mediated gene transfer technique (in vivo sperm precursors)

Other methods for creating transgenic spermatozoa have also been explored. One of these approaches is testis-mediated gene transfer (Fig. 7), which is a simplified form of SMGT because it does not involve IVF or embryo transfer. In addition, the testis is regarded as an immune-privileged organ. The ability to transfer genes into specific testicular cell types in vivo should provide a tool for studying the molecular regulation of spermatogenesis [42]. The process of gene transfer into epididymal spermatozoa by a DNA-transfectant complex injected into the testis is being investigated. Foreign DNA inserted into the testis, on the other hand, is thought to be quickly transferred to the epididymal ducts via the rete testis and efferent ducts, where it is integrated by epididymal epithelial cells and epididymal spermatozoa [43].

Adenovirus vector solution injected into the interstitial space (intratesticular injection) or seminiferous tubules (intratubular injection) of the mouse testis is another method for introducing foreign genes. The results suggest that adenovirus-mediated gene transfer may be effective for transfecting testicular somatic cells, and that this approach may be applicable for in vivo gene therapy for male infertility in the future, despite the fact that spermatogenesis is slightly impaired and the inflammatory response caused by these methods may present some problems. The findings also suggest that TMGT could be used for fetal gene therapy and the production of transgenic animals in general [44].

## Somatic cell nuclear transfer (SCNT)

**Fig. 8:**
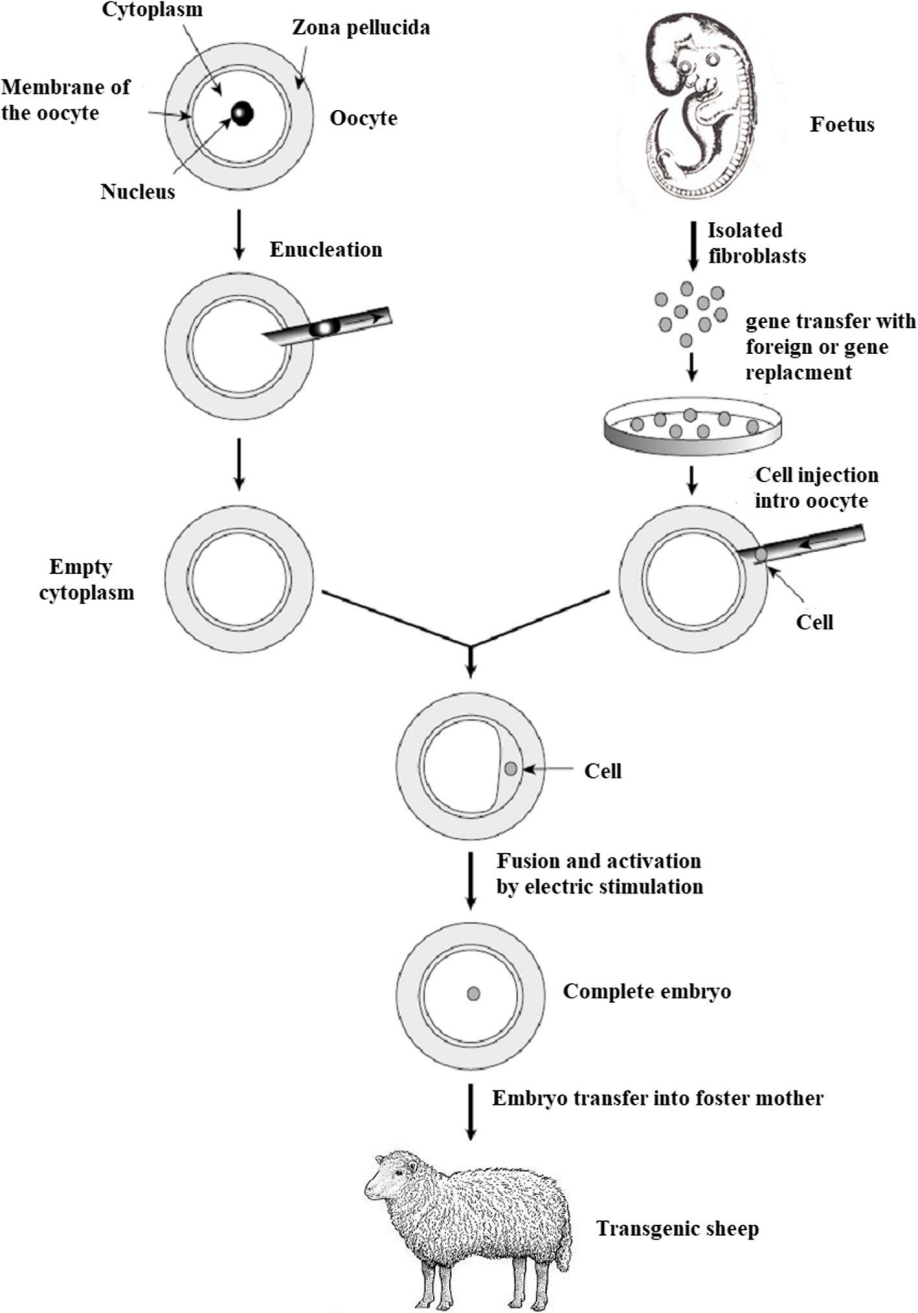
The somatic cell nuclear transfer technique

The nuclear transfer offspring development is an inefficient method and the successful percentage ranged from 0.5 and 5.0%. Losses happen during pregnancy, at birth, and in the weeks and months afterward, and a number of developmental anomalies have been documented. The causes of these abnormalities are unknown, however, they could be caused by improper or insufficient reprogramming or even issues with imprinted genes [45–47]. It may be possible to clarify the mechanisms behind these processes by having a better grasp of the systems controlling normal development. The technique involves the transfer of a somatic cell nucleus to an enucleated egg’s cytoplasm where it will be reprogrammed by egg cytoplasmic components to become a zygote [48–50]. In mammals, the zygote needs to be artificially implanted into a surrogate mother’s uterus [51, 52]. Willadsen had his first significant success with SCNT in 1986, when he produced lambs cloned from embryo nuclei at stages ranging from 8 to 16 cells. This discovery piqued the interest of researchers in using nuclear transfer to multiply embryos derived from high-value agricultural animals [53]. This time-consuming procedure also opened up new and exciting prospects for animal transgenesis. When the nuclei utilized in embryo reconstruction come from a cell with some genetic modification, the animals created by nuclear transfer could be regarded as a group of transgenic animals (addition, substitution, or alteration of some gene). In this view, transgenic embryos and animals are defined as those produced via nuclear transfer of genetically changed cells, as they carry the initial changes present in the nucleus of the donor cell from which the animal was derived (Fig. 8).

Interest-specific exogenous genes can be transfected into somatic cells and then transferred to pluripotent cells (cells of morulae or blastocysts). The progeny of the chimera can inherit the exogenous gene, making them transgenic [27]. Cultivated cells can be transfected in this fashion, and the insertion and expression of the transgene can be validated before using these cells to produce genetically modified cloned animals [54]. In this procedure, the DNA is randomly incorporated into the genome by selection pressure; however, the transgenic cells may be completely described (integration site, number of integrated copies, and transgene integrity) before being used for nuclear transfer. As a result, while “reconstructed” nuclear transfer (NT) embryos have a decreased developmental capability, the vast majority of animals born are transgenic, making this approach far more efficient than pronuclear microinjection. Transgenesis efficiency has increased considerably, thanks to somatic cell nuclear transfer. Only somatic cells, which are utilized to create genetically engineered animals, can perform gene substitution through homologous recombination at the moment. Gene inactivation has been achieved in sheep [55] and pigs [56]. The majority of animals cloned from transfected somatic cells express the transgene, according to results observed in cattle, sheep, goats, and pigs.

## Applications of transgenic animals

### Animal production

Some of the practical uses of transgenesis in animal production include greater prolificacy and reproductive performance, higher feed consumption and growth rate, improved carcass composition, improved milk production and/or compositions, and increased disease resistance (Table 1). The most important candidate gene for generating GH-transgenic farm animals to increase the growth rate and milk production [2–4] is growth hormone. Myostatin is a negative regulator of muscle cell proliferation during fetal development. It is know that inactivating mutations in a number of species, including cattle, develop a muscle overgrowth phenotype [57]. Although the increased muscle can be considered a positive trait, the increase in dystocia related to the size of the calves at birth limit the utilization of these naturally occurring myostatin mutations in production agriculture.

Genetically modifying animals to make their organs immunologically compatible for use as human transplants or to improve commercial recombinant protein output in the transgenic mammary gland was the first animal-focused experiment [58].

**Table 1:** An examination of transgenic livestock species and their value in animal production

Animal	Genes introduced or deleted	Performance criteria (consumer benefit)	Reference
Bovine	*β* and *κ* casein	Casein protein expression has increased (improved protein content of milk)	[59]
Bovine	Intestinal lactase	Lactose in milk is being reduced (lactose intolerant people)	[60]
Bovine	Lysostaphin	Resistance to mastitis (reduced use of antibiotics)	[61]
Bovine	*β*-Lactoglobulin	Higher milk production of this protein, as well as increased growth and illness resistance in milk-fed calves (reduced antibiotic use and improved health benefits)	[62]
Ovine	Growth hormone	Increased growth rates, improved feed conversion efficiency, lower carcass fatness, and higher lactation rates (leaner meat)	[63]
Ovine	Myostatin	In sheep, myostatin expression was reduced, and muscle mass was raised (leaner meat)	[64]
Porcine	Insulin-like growth factor 1	Increased growth rate and lower fat content in the carcass (leaner meat)	[65]
Porcine	*α*-Lactalbumin	Piglets’ growth rate has increased, and their health has improved	[66]

**Fig. 9:**
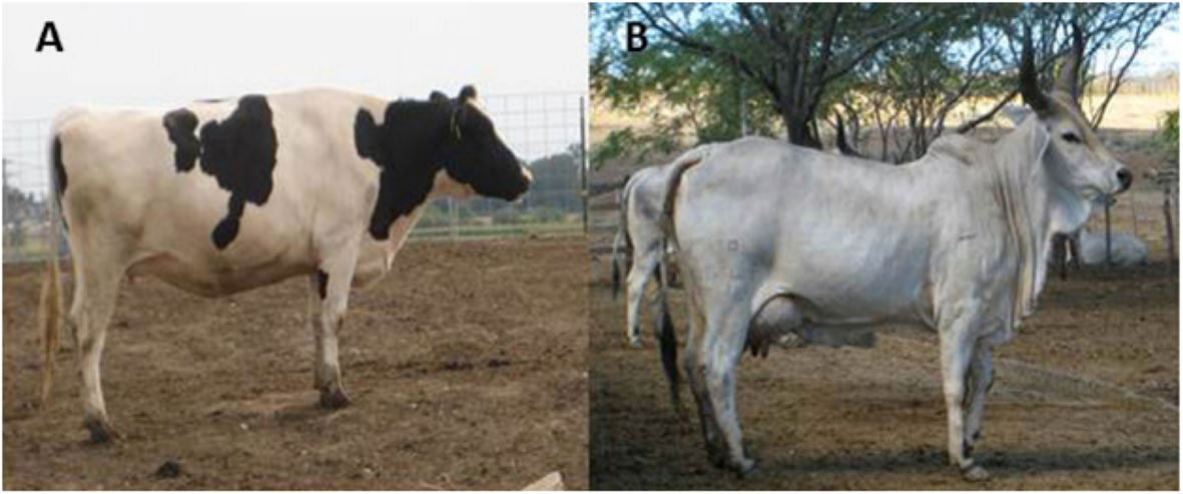
Can transgenic technology produce comparable milk volume? Small improvements in milk volume in Guzerat cows (left) using genetic material from high-producing Holsteins (right) could have a significant impact on Brazilian beef production [68]

Transgenic technology advancements offer the chance to modify milk's composition or manufacture whole new proteins in milk. It is possible to increase livestock growth or survival by changing the composition of milk. To do this, transgenic animals must be developed that: (1) produce more milk; (2) produce milk with higher nutrient content; or (3) produce milk with a useful "nutriceutical" protein. Lactose, fat, and protein are the three main nutrients in milk. We can influence the development and well-being of the growing offspring by improving any one of these factors. Increased milk yield or composition can be advantageous for cattle, sheep, and goats raised for meat production [67]. Heat-tolerant livestock breeds, like Bos Indicus cattle, are necessary for the increase of agricultural productivity in tropical areas. Bos Indicus cow breeds do not, however, yield a lot of milk. Weaning weights in Brazilian cattle of the Nelore or Guzerat breeds may be significantly increased by increasing milk production to just 2-4 litres per day (Fig. 9). Improvements in weaning weights in meat-type breeds like the Texel sheep and Boer goat can be compared in a similar way. By using transgenic technology in this way, offspring may grow and survive better [68].

Lactose synthesis and milk volume [67, 68] are both aided by alpha-lactalbumin. The milk protein bovine alpha-lactalbumin was overexpressed in transgenic homozygous sows, resulting in up to 0.9 g of bovine alpha-lactalbumin per liter of milk produced by the sow. Weight gain was increased in piglets sucking alpha-lactalbumin gilts (days 7–21 after parturition). As a result, increased milk protein expression in transgenic sows may aid pig lactation success. Furthermore, transgenic cows with extra copies of the bovine beta- and kappa-casein genes produced milk with 8–20% higher beta-casein levels and twice the kappa-casein levels. This work demonstrates that by employing a transgenic approach to improve the functional qualities of dairy milk, a main component of milk in high-producing dairy cows may be dramatically altered.

The transgenic sheep with wool keratin and keratin-associated protein (KAP) genes could be utilized to alter the protein composition of wool fibers, resulting in fiber types with improved processing and wearing qualities [69]. Aquaculture transgenic research is rapidly developing on a global basis. Fish and shellfish have a high fertility rate. Fertilization is frequently easy, and fertilized eggs develop outside the body, requiring no major manipulation, such as preimplantation. As a result, making transgenic fish or shellfish is rather straightforward. In Australia, the focus is on the possible use of transgenesis to control wild populations such as European carp.

### Environmental pollution

Phytic acid is a compound that pigs cannot produce naturally. On the other hand, 50 to 70% of the phosphorus in grain comes from phosphorus. As a result, many farmers must use an enzyme called phytase to enhance pig diets. Phytase helps pigs consume more of their nutrition by breaking down phytic acid. Farmers pay a high premium for the phytase enzyme, and it might be damaged or destroyed accidentally when they mix feed. To address this issue, the Enviropig was developed.

Enviropigs have salivary glands that have been genetically engineered to aid in the digestion of phosphorus in feedstuffs and reduce phosphorus pollution in the environment [5]. The salivary glands of the transgenic pig synthesized phytase, removing the need for extra supplements or enzymes in the feed. The Enviro-Pig produces less phosphorus in its face by consuming more phosphorus [70].

### Medicine

#### Nutritional supplements and pharmaceuticals production

Milk composition can be altered in several ways: by changing the concentration of unsaturated fatty acids, reducing the lactose content, removing *ß*-lactoglobulin, or combining nutraceuticals in milk. By combining nutritional and genetic interventions, researchers are now hoping to develop “medicine milk,” rich in specific milk components that have implications for health as well as treatment. In 1997, the first transgenic cow, Rosie, produced human alpha-lactalbumin-enriched milk at 2.4 g per liter. This transgenic milk is a more nutritionally balanced product than natural bovine milk and could be given to babies or the elderly with special nutritional or digestive needs [71]. This transgenic milk is a more nutritionally balanced product than natural bovine milk and could be given to babies or the elderly with special nutritional or digestive needs.

#### Xenotransplantation

Human life expectancy is increasing, and doctors' abilities to transplant patients' cells and organs are also improving. Patients who require transplants are increasing, while organ donors are growing far more slowly [72]. Xenotransplantation is the term used to describe the transfer. The gap between the availability and demand for human organs is a barrier to clinical transplantation [73, 74]. Every day, almost 17 people pass away while awaiting an organ transplant. According to the US Government Information on Organ Donation and Transplantation, more than 106,941 people were on the transplant waiting list as of October 2021, whereas only 39,000 transplants were carried out in 2020 [data available at URL: https://www.organdonor.gov/statistics-stories/statistics.html (accessed September 2021)]. Xenotransplantation might be a good solution to this major issue. Although the surgical part of the procedure was frequently successful, the xenogeneic foreign organs were always violently rejected and killed. As a result, the technology aims to create humanized organs from pigs by preventing organ rejection brought on by physiological differences between humans and pigs as well as the spread of illnesses with genetic origins [75, 76]. A pig protein that can lead to donor rejection currently limits the utilization of xenotransplantation, although research is being done to substitute the pig protein with a human protein [67]. Concerns with welfare, ethics, and clinical and safety considerations are additional difficulties. Future solutions to the issue of transgenic organs may involve improving the supply of National Health Services and promoting tissue donation and stem cell regeneration [76].

#### Pharmaceutical animals

A gene encoding a pharmaceutically essential protein can be isolated, inserted into an expression vector, and then delivered into cells or organisms that produce the protein in high quantities. Human insulin was created from genetically modified bacteria. The vast majority of diabetics now receives recombinant insulin rather than derived pig insulin. The recombinant hormone is purer and structurally equivalent to native human insulin.

For more than a decade, the only type of human growth hormone that has been used is one generated from bacteria. As a result, the risk of contamination by the human prion that causes Creutzfeldt-Jakob disease has been eliminated [77]. This technique, while effective, quickly reveals its limitations. Some proteins are difficult for bacteria to synthesize. Others are difficult to purify because they become insoluble in bacteria. Furthermore, many pharmaceutically essential proteins, especially human proteins, are glycosylated or require posttranscriptional alteration in order to function physiologically. Bacteria and recombinant yeast are unable to progress through the majority of these stages.

Genetically engineered animal cells are being exploited as a source of recombinant proteins. It became possible to create transgenic farm animals larger than mice (Table 2) (rabbits, pigs, and sheep) that secrete foreign proteins in their blood, milk, and other bodily fluids. Milk protein gene promoters were fused to the coding area of the sheep *β*-lactoglobulin gene, as well as human tissue plasminogen activator to the coding *β*-lactoglobulin gene [78]. In an experimental setting, this method allowed the secretion of 100 foreign proteins in milk; milk with higher casein levels, which is good for making cheese, or milk with particular qualities to fill population gaps, such as lactose-free milk for the Asian market, milk without β-lactoglobulin for consumers with allergies, or milk containing the human β-lactoferrin protein to ensure the health of newborns [79]. A number of them are detected in large amounts in rabbit, sheep, goat, and cow milk and are clinically tested. Human a-glucosidase, generated from rabbit milk, was one of the proteins that improved the clinical state of babies with Pompe disease. Collagen, fibrinogen, spider silk, and EC superoxide dismutase are just a few of the complex foreign proteins that may be produced by the mammary gland [78].

**Table 2:** Some prescription drugs created by transgenic animals

Drug	Disease/target	Animal	Reference
Alpha-lactalbumin	Anti-infection	Cow	[80]
Human protein C	Thrombosis	Pig, sheep	[81]
Fibrinogen	Wound healing	Cow &sheep	[82, 83]
Glutamic acid decarboxylase	Type 1 diabetes	Mouse, goat	[84]
Human serum albumin (HAS)	Maintains blood volume	Mouse, cow	[85]
msp-1	Malaria	Mouse	[86]
CFTR	Cystic fibrosis	Sheep, mouse	[87]
Human calcitonin	Osteoporosis	Rabbit	[88]
Lactoferrin	Tract infection, infectious arthritis	Cow	[89]

#### Industry

Nexia Biotechnologies Inc. has developed a strain of dwarf goats from West Africa that naturally breed and lactate early (BELE®), decreasing transgenic protein production time compared to sheep, cows, and conventional goats. Male BELE® goats, for example, reach sexual maturity at the age of 15 weeks, whereas traditional male goats reach sexual maturity at the age of 30 weeks. This reduces the time it takes to produce a transgenic herd. Clinical trials and product commercialization can start sooner due to the shorter time between lab amounts and production quantities of protein [52]. Two Canadian scientists spliced spider genes into the cells of goats in 2001. The goats began to make silk with their milk, which is the strongest material in the world and named “Bio-Steel.” Scientists can manufacture a light, durable, and flexible material by separating polymer strands from milk and weaving them into thread, which might be utilized in military uniforms, medical micro sutures, and tennis racket strings, among other applications [48]﻿.

#### The risks of the application of transgenic animals

The main environmental issue associated with transgenic animals is the possibility of their escape. The risks differ significantly depending on the transgene and the species. Some farm animals cannot live in the wild because they are confined. Transgenic animals are mostly used to study genes and biological functions. Transgenic animals might also be a useful model for investigating human and animal diseases and testing experimental medication or a source of human organs [90]. The technology involved in the production of transgenic animals holds great promise for both agriculture and biomedicine but also has potential risks. So, it is important for scientists to become engaged in the discussion and consideration of ethical issues and concerns surrounding the implementation of this work and the effects on the environment, farmers, and consumers; the use of this technology is not simple, efficient, or inexpensive [91]. The "biosafety" field of research to manage the ecological effects of transgenic animals has recently emerged [90]. Due to the high cost of the transgenic process, we can only obtain a small number of animals. Because of this, backcrossing is a difficult process that must be used to reintroduce such animals into the herd. To make this process economically feasible, it is essential to possess in-depth knowledge of the most recent advancements in assisted reproduction techniques, such as artificial insemination combined with embryo transfer or in vitro embryo formation [79, 92]. The only real issue with using transgenic animals to study human diseases is ethical. The same is true for animals used as sources of proteins or organs for pharmaceuticals because the manipulation of embryos can have a negative impact on animal welfare [93, 94]. Commissions with extensive experience in this area with conventional chemical medications are evaluating the medical issues caused by the usage of pharmaceutical proteins [72, 95]. There is probably no damage to the environment from transgenic animals. More dangers may be posed by transgenic fish and live virus-based vaccinations, which raise complex issues for environmental risk assessment [96]. According to Muir, and Howard, [97], growth hormone (GH)-transgenic fish are rapidly growing and more sexually mature but are more fragile and have shorter lifespans than the controls. The quickly expanding transgenic fish that was released into the ocean afterward could be to blame for the local extinction of the species. Although unlikely, this prospect cannot be discounted, and the regulatory bodies have not, up until this point, approved the breeding of fast-growing fish using the current methods. The issue could be resolved by sterilization of females and breeding of females only or completely isolating fish farms. Biosafety organizations, on the other hand, may allow humans to consume rapidly growing fish but not reproduce them. Assessing the impact of transgenic animals on biodiversity requires knowledge. So that the state can evaluate the impacts of potential transgenic animals on biodiversity, this information must be updated on a regular basis [97].

## Conclusion

Genetic engineering is used to incorporate foreign genes into the animal genome so that they can be inherited and expressed by offspring via transgenic animal technologies. To address the current and future demands of the human race, transgenic animals are required in agricultural techniques, food supply development, and food consumption management. Moreover, with the development of disease-resistant animals and other approaches for enhancing animal production capacity, animal transgenesis has the potential to replace traditional drug use in the future. It was also used to improve human health by filling organ shortages and producing vital pharmaceuticals to treat human illnesses. Animal welfare and ethics are major factors that make technology adoption difficult. Transgenesis’ efficiency needs to be improved, and awareness should be raised to avoid strong opposition to such novel technologies.

## Data Availability

Not applicable.

## Abbreviations

CP: Crude protein
CF: Crude fiber
EE: Ether extract
TDN: Total digestible nutrient
DCP: Digestible crude protein
DMI: Dry matter intake
LBW: Live body weight
TBWG: Total body weight gain
ADG: Average daily gain
TDNI: Total digestible nutrient intake
DCPI: Digestible crude protein intake
AIA: Acid insoluble ash
OM: Organic matter
NFE: Nitrogen-free extract
NDF: Neutral detergent fiber
ADF: Acid detergent fiber
ADL: Acid detergent lignin
